# Editorial: Forest Health Under Climate Change: Effects on Tree Resilience, and Pest and Pathogen Dynamics

**DOI:** 10.3389/fpls.2019.01157

**Published:** 2019-10-15

**Authors:** Riikka Linnakoski, Risto Kasanen, Aikaterini Dounavi, Kristian M. Forbes

**Affiliations:** ^1^Forest Health and Biodiversity, Natural Resources, Natural Resources Institute Finland (Luke), Helsinki, Finland; ^2^Department of Forest Sciences, University of Helsinki, Helsinki, Finland; ^3^Department of Forest Protection, Forest Research Institute Baden-Württemberg, Freiburg, Germany; ^4^Department of Biological Sciences, University of Arkansas, Fayetteville, AR, United States

**Keywords:** tree stress, forest health, forest pests, forest pathogens, disease

## Introduction

Climate change is having important effects on forest dynamics, which can be both positive and negative for natural and managed ecosystems. To date, most empirical and synthesis research has focused on climate change implications for tree species distributions and productivity, while the potential impacts on forest pests and pathogens and the effects on tree population health have been comparatively neglected. These present some of the greatest threats to global forest health under climate change. Climate change is altering the distributions and population structures of forest pests and pathogens, the way they interact with trees, and their evolutionary capacity, while also affecting the capacity of forest systems to resist and tolerate attacks.

Our ability to predict the impacts of interactions among biotic agents and climate change is currently very limited and has important implications for managing increasingly disturbed forests (both natural forest ecosystems and plantation forestry). Accumulating research of environmental effects on interactions among trees, pests, and pathogens suggests that there is an urgent need for systems-based research to investigate both longstanding and novel biotic interactions. More specifically, it is crucial to record changes in forest pest population dynamics, address the factors that trigger their outbreaks, and analyze the development of forest tree disease epidemics, as well as changes in tree susceptibility and resilience under a changing climate. Recent research has started to address these questions, and this Research Topic aims to contribute to the emerging field of climate change research.

In line with this objective, articles in this special issue fall under three broad topics: (1) tree responses to stress associated with climate change; (2) associations among climate, trees, and insect herbivory; and (3) forest pathogen outbreaks and disease spread under a changing climate.

### Tree Responses to Stress Associated With Climate Change

Varsamis et al. tested the adaptive potential of beech seedlings from two provenances in northeast Greece, which vary in the temporal distribution of precipitation. Seedling survival, growth, and leaf phenological traits were analyzed under simulated climate change conditions in a growth chamber; temperature and precipitation levels predicted for the year 2050 were applied for 3 years under two different irrigation schemes. Comparisons were also made between seedlings grown under field conditions. The study found that beech seedlings were generally able to survive under predicted climate change conditions. Differences in growth and leaf phenological traits occurred among treatment groups, but these varied among years and were sometimes absent. In a related study, Kohler et al. investigated the effects of soil liming on the drought tolerance of Norway spruce. Radial increment in relation to drought was compared over 30 years between limed and nonlimed sites. The study found no direct improvement of drought tolerance resulting from liming. However, positive effects were observed in one site treated with wood ash 30 years after the first lime treatment, and growth recovery and resilience after severe drought were marginally enhanced by liming. According to the results, spruce trees growing in limed soils faced a shorter period of stress and are consequently more resilient to pest and pathogen attacks.

### Associations Among Climate, Trees, and Insect Herbivory

A study by Whipple et al. used long-term monitoring data to examine interactions among poor soil, severe drought, and moth herbivore susceptibility in a pinyon pine tree species that has suffered extensive climate change–related mortality. Contrary to the authors’ hypothesis, they found that chronically stressed and herbivore-susceptible trees had smaller declines in performance during drought years than less-stressed trees. Tree mortality was also higher in finer soils, and moth abundance declined during droughts. The authors concluded that the results support the notion that stressed trees have adapted or acclimatized to harsh conditions, rendering them less sensitive to drought and herbivore effects. In another moth herbivory study, Camarero et al. assessed the effects of climatic stress and European gypsy moth (*Lymantria dispar dispar*) outbreaks on wood characteristics of radiata pine and chestnut tree populations in Spain. Radial growth decreased substantially in both tree species because of defoliation. Climate explained the majority of growth variability in defoliated chestnuts. However, radial growth and wood density were important for radiata pine, and the authors suggest these variables could be used as indicators for susceptibility to defoliation by the gypsy moth. Hentschel et al. performed an extensive analysis of ecological variables related to past outbreaks by three insect defoliators of Scots pine in Germany. They found that the dynamics of these herbivores were influenced by environmental variables in important but different ways. For example, *Lymantria monacha* feeding was mainly dependent on the tree stand surroundings, whereas *Diprion pini* was largely driven by climate, and *Dendrolimus pini* followed a mixed pattern determined by climatic and forest structure parameters.

Three studies examined genetic aspects of host resistance to climate changes and herbivore attack. Stone et al. sought to determine if susceptibility to herbivory influenced the diversity and composition of the extended community of arthropod species, using pinyon pine in the southwestern United States, which shows genetic-based resistance and susceptibility to pinyon needle scale. Among several findings, they reported that during extreme drought, arthropod community composition on scale-excluded trees resembled susceptible trees, indicating composition was strongly influenced by tree genetics independent of tree architecture. However, under moderate drought, community composition on scale-excluded trees resembled resistant trees, indicating traits associated with tree architecture became more important. Creyaufmüller et al. investigated interactions between oak seedlings from two distinct regions in Germany and root herbivory by cockchafer larvae, which can enhance the impacts of drought. Both oak populations were genetically variable, but no clear genetic pattern for feeding preference emerged. Contrary to field observations, larvae preferred seedlings from the source area where severe damage by cockchafer does not occur. Larvae feeding preference was thought to be driven by root-released volatile terpenes and benzenoids, and the authors identified five oak terpene synthase genes with geographically structured expression patterns. Finally, Six et al. tested whether a genetic basis is responsible for certain individual mature white bark and lodgepole pine trees in western North America surviving recent climate-driven outbreaks of the mountain pine beetle. They compared sequence repeats from surviving trees to those of the general tree populations and identified segregation among these tree groups that supports a genetic basis to survivorship.

### Tree Pathogen Outbreaks and Disease Spread Under a Changing Climate

Two studies evaluated pathogen outbreaks in northern Europe, one of the areas where climate change effects are expected to be most pronounced. Brodde et al. reconstructed the development of the first large outbreak of *Diplodia* tip blight in Scots pine in northern Europe, which occurred in Sweden in 2016. They found that isolated attacks began approximately 10 years earlier and culminated in 90% of trees being attacked in 2016. Limited damage occurred in surrounding stands, suggesting the pathogen was a new introduction. Warm temperatures were associated with greater tree damage and slow growth, while cold and rainy conditions supported tree growth and impaired attacks. Significantly, the study identified no restrictions to *Diplodia* tip blight becoming a serious pathogen of northern European forests. In a similar study, Hietala et al. examined the increase of propagule pressure of *Hymenoscyphus fraxineus*, a fungus from Asia causing the dieback of European ash, by monitoring its sporulation in a newly infested stand in Norway. Interactions with a competing nonpathogenic native fungus, *Hymenoscyphus albidus*, were also investigated. The authors found that infection pressure was strongly influenced by summer temperatures (higher temperatures favor population growth) and that *H. fraxineus* populations grew exponentially during years with favorable local conditions.

In southern Italy, Colangelo et al. investigated the effects of drought and *Phytophthora* on populations of two declining oak species (*Quercus cerris* and *Quercus pubescens*) using comparisons with nearby nondeclining trees. The growth rate of both tree species decreased with drought and warming temperatures and was most pronounced in *Q. cerris*. No effects of *Phytophthora* on tree performance were identified, with the fungus being more prevalent in nondeclining trees. Pan et al. assessed the virulence of three blue-stain fungal associates of pine shoot beetles on Yunnan pine. These fungal species are associated with *Tomicus* shoot beetles, which often kill Yunnan pine trees in China. Their results show that all three blue-stain fungi (*Leptographium wushanense*, *Leptographium sinense*, and *Ophiostoma canum*) are potentially pathogenic to Yunnan pine, with *L. wushanense* and *L. sinense* more virulent than *O. canum*. The authors stated that the two *Leptographium* species may facilitate bark beetle colonization and contribute to their aggressiveness and, given sufficient spread, could cause significant damage to Yunnan pine forests across southwest China. Lastly, Linnakoski and Forbes extended this concept in an opinion article broadly discussing the role of fungal symbionts of wood-boring insect pests and their ability to both inflict their own damage on trees and seedlings and to enhance the severity of insect damage. These implications are likely to change and potentially be amplified under climate change as insect vectors invade new areas. The authors highlight how little we understand the effects of fungal pathogens in comparison to their insect vectors and outline gaps in knowledge to help advance this topic, including the need for baseline information of insect-fungal associations in their native ranges and for assessment of fungi roles in tree declines that are usually attributed to their more conspicuous insect vectors.

Overall, our understanding of the effects of climate change on forest pest and pathogen dynamics is in its infancy, with generalizations extremely difficult due to the huge amount of diversity in study systems and climate change effects across geographical regions. Forests are critical ecosystem components across many global regions, and a human capacity to predict and respond to pest and pathogen outbreaks in these habitats as environments continue to change is clearly required. We hope that the work presented in this Research Topic will help to both consolidate the field and motivate researchers to address the many gaps in knowledge.

## Author Contributions

All authors listed have made a substantial, direct, and intellectual contribution to the work and approved it for publication.

## Conflict of Interest

The authors declare that the research was conducted in the absence of any commercial or financial relationships that could be construed as a potential conflict of interest.

